# Anemia secondary to valproic acid therapy in a 13-year-old boy: a case report

**DOI:** 10.1186/1752-1947-6-239

**Published:** 2012-08-10

**Authors:** Barbara Kaczorowska-Hac, Agnieszka Matheisel, Lucyna Maciejka-Kapuscinska, Jakub Wisniewski, Anna Alska, Elzbieta Adamkiewicz-Drozynska, Anna Balcerska, Iwona Reszczynska

**Affiliations:** 1 Department of Pediatrics, Hematology, Oncology, and Endocrinology, Medical University of Gdansk, Gdansk, Poland; 2 Department of Developmental Neurology, Medical University of Gdansk, Gdansk, Poland

**Keywords:** Valproic acid, Anemia, Child

## Abstract

**Introduction:**

Valproic acid is a commonly used anti-epileptic drug. Hematological toxicities are among the occasionally observed adverse effects of this medication.

**Case presentation:**

We present the case of a 13-year-old Caucasian boy who demonstrated mild anemia 12 months after the introduction of valproic acid therapy. A bone marrow biopsy revealed maturation arrest of proerythroblasts.

**Conclusion:**

Prompt diagnosis and valproic acid discontinuation resulted in the patient’s recovery.

## Introduction

Aplastic anemia, pure red cell aplasia, macrocytosis, leukopenia and thrombocytopenia are some of the hematological adverse effects of valproic acid therapy cited in the literature [1,2]. Some of these effects can lead to life-threatening complications. We present the case of a 13-year-old Caucasian boy who presented with mild macrocytic anemia 12 months after the introduction of valproic acid therapy. A bone marrow biopsy revealed maturation arrest of proerythroblasts. Spontaneous recovery was observed after the patient’s therapy was discontinued.

## Case presentation

A 13-year-old Caucasian boy admitted to the Hematology Department at our institution was diagnosed with anemia. Eight years prior to the admission he had been diagnosed with epilepsy. He was treated with 10 mg lamotrigine per day, which was withdrawn after one year because the seizures had ceased. Six years after the anticonvulsant therapy was discontinued a review electroencephalogram (EEG) revealed many synchronic epileptiform changes, that is, sharp waves and spikes. In spite of the fact that there were no reported seizures during the above-mentioned period, 20mg/kg/day valproic acid therapy was introduced.

After 12 months of therapy, during a routine visit, the neurologist noticed the patient presented with pallor. He did not report any other ailments and was leading an active life. His laboratory parameters were as follows: hemoglobin (Hb), 9.2g/dl; red blood cells (RBCs), 2.71 × 10^12^/L; mean corpuscular volume (MCV), 100 fl; mean corpuscular hemoglobin (MCH), 33.9pg; mean corpuscular hemoglobin concentration (MCHC), 34g/dl; platelets, 321 × 10^9^/L; and white blood cells (WBCs), 4300 × 10^6^/L. The only other medication taken by the patient was loratadine for his pollen allergy. Both the patient and his mother denied any exposure to environmental toxins, taking other medications or drugs, drinking alcohol, or smoking cigarettes. They confirmed full adherence to the therapy. The patient also denied having had any respiratory tract infections in the period preceding examination.

**Figure 1:**
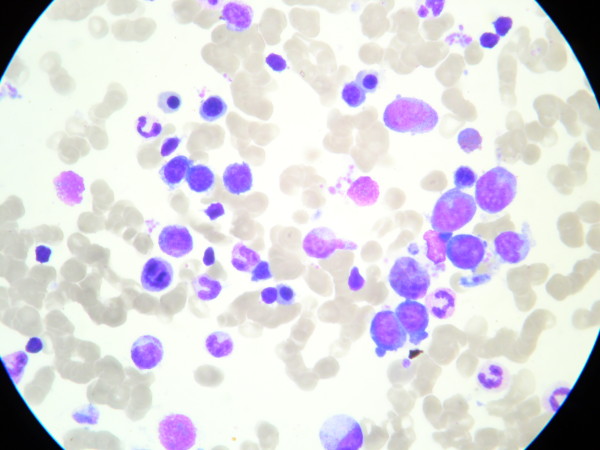
Normocellular bone marrow with mild dyserythropoiesis: diminished number of orthochromatic erythroblasts.

Although pale at the time of admission, the boy was in relatively good condition and there were no other abnormalities in the physical examination. Laboratory tests confirmed macrocytic anemia. Serum iron and erythropoietin concentration were elevated, but other biochemical measurements were within the normal range (Table 1). Serological tests excluded cytomegalovirus, Epstein-Barr virus, hepatitis B and C, parvovirus B19, and toxoplasmosis infections. Antinuclear antibodies were negative, serum valproate concentration was within the reference range, and urine cultures were negative. A bone marrow biopsy revealed normocellular bone marrow with a regular myeloid:erythroid ratio and megakaryocytes and without malignant cells or megaloblastic changes. Mild dyserythropoiesis was observed: The amount of proerythroblasts was regular (5%), but the number of orthochromatic erythroblasts was diminished (3.3%) (Figure 1). Because we suspected that valproic acid could have induced the patient’s anemia, we gradually suspended it under close neurological control. On the EEG examination, only a few slow waves were found in the frontal region.

**Table 1:** Laboratory findings of the patient at the admission

**Parameter**	**Value**	**Normal range**
Hemoglobin	9.0g/dl	14 to 18g/dl
Red blood cells	2.76 × 10^12^/L	4.7 to 6.1 × 10^12^/L
Hematocrit	26.7%	42% to 52%
Reticulocytes	1.8%	0.5% to 2.0%
Mean corpuscular volume	96.7fl	79 to 95fl
Mean corpuscular hemoglobin concentration	33.7g/dl	33 to 38g/dl
Mean corpuscular hemoglobin	32.6pg	27.5pg
White blood cells	4520 × 10^6^/L	4500 to 11,000 × 10^6^/L
Neutrocytes	1.69 × 10^6^/L	1.9 to 8.0 × 10^6^/L
Platelets	311 × 10^9^/L	130 to 400 × 10^9^/L
Iron	185μg/dl	31 to 144μg/dl
Ferritin	73.73ng/ml	21.81 to 274.66ng/ml
Vitamin B_12_	426pg/ml	189 to 883pg/ml
Folic acid	7.9ng/ml	2.34 to 17.56ng/ml
Creatinine	0.58mg/dl	0.5 to 1.0mg/dl
Alanine aminotransferase	17U/L	0 to 55U/L
Aspartate aminotransferase	20U/L	5 to 34U/L
Lactate dehydrogenase	258U/L	125 to 220U/L
C-reactive protein	0.7mg/L	0.0 to 5.0mg/L
Erythropoietin	1257mU/ml	8 to 36mU/ml
Valproate	71mg/L	50 to 100mg/L
Fetal hemoglobin	1.06%	<1%

**Figure 2:**
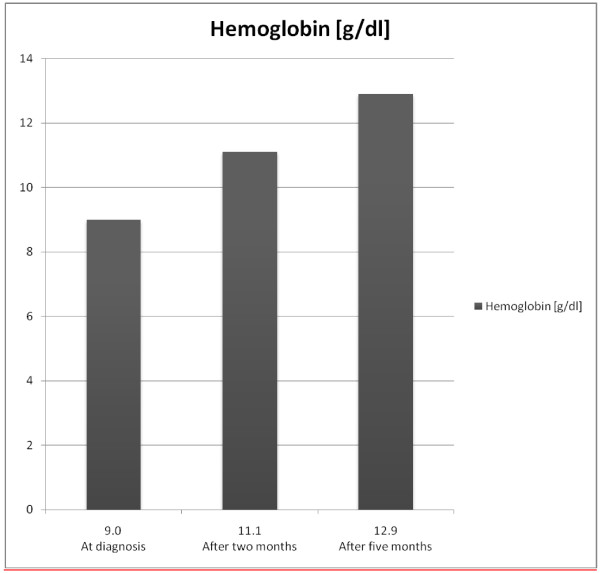
Increase in hemoglobin after valproic acid suspension.

During the observation period, morphology tests improved systematically (Figure 2), and after 5 months they were as follows: Hb, 12.9g/dl; RBCs, 4.47 × 10^12^/L; MCV, 85.9fl; MCH, 28.9pg; MCHC, 33.6g/dl; reticulocytes, 0.4%; platelets, 226 × 10^9^/L; WBCs, 4800 × 10^6^/L; and neutrocytes, 1750 × 10^6^/L.

The patient is still without anticonvulsant therapy and does not have any neurological symptoms.

## Discussion

Valproic acid is a widely used anti-convulsant agent and is also clinically effective as a mood stabilizer in the treatment of manic depression. However, it may induce a variety of side effects, such as bone marrow toxicity, which may lead to life-threatening complications. Severe hematological complications such as thrombocytopenia, leukopenia, aplastic anemia, and pure red cell aplasia have been described [3-6]. Several cases of these adverse effects have been noted in the literature. Nasreddine *et al*. [7] reported that, of 851 patients who were treated with valproic acid, 17.7% experienced at least one episode of thrombocytopenia. Rahman *et al*. [8] reported that, of 131 children and adolescents treated with valproic acid, 26% experienced leukopenia. Although Handoko *et al*. [4] reported that the use of valproic acid is associated with a ninefold risk of aplastic anemia, Zaccara *et al*. [2] confirmed that the incidence of pure red cell aplasia is sporadic.

Our 13-year-old patient was on a standard dose of valproic acid for merely 12 months. He reported none of the above-described ailments and did not present with any abnormalities except for the marked pallor. Mild macrocytic anemia was confirmed by a routine blood test. Common causes of this condition, such as vitamin B_12_ and folic acid deficiency, were excluded. There was no evidence of valproate overdose, and tests for infections were negative. Moreover, there was no evidence of any influence of loratadine on the development of anemia. The bone marrow biopsy showed a diminished number of orthochromatic erythroblasts, but proerythroblasts were within the normal values. The myeloid:erythroid ratio was within the reference range. The patient’s recovery was spontaneous after the discontinuation of the medication, and his blood parameters improved over a five month observation period.

When we reviewed the literature, we found that the population of patients stricken with valproic acid hematological complications seemed to be heterogenic. Some of the patients previously documented had undergone relatively longer treatment [1]. Some of them had been treated with higher doses of valproate [9], and some had been taking other anticonvulsants [10] or other additional medications [11]. Our patient presented with normocellular bone marrow with qualitative abnormalities of erythropoiesis, whereas other authors have presented cases of patients with severe abnormalities of the bone marrow, such as hypocellularity, myelodysplasia, and changes resembling promyelocytic leukemia as well [1,3,12]. Thus, we believe that valproate caused dyserythropoiesis in our patient, resulting in mild macrocytic anemia.

It has previously been confirmed in a microarray analysis by Chateauvieux *et al*. [13] that valproic acid is able to alter hematopoiesis by inhibition of erythroid differentiation in the experimental K562 cell lineage. We suspected that the anticonvulsant had a similar impact on erythroid precursors in our patient. This led to the decision to withdraw the valproate, resulting in a successful and lasting outcome.

## Conclusion

Neurologists should be aware of the hematological adverse effects of valproic acid. Regular testing of hematological parameters is essential in patients on such treatment.

## Consent

Written informed consent was obtained from the patient’s legal guardian for publication of this manuscript and accompanying images. A copy of the written consent is available for review by the Editor-in-Chief of this journal.

## Competing interests

The authors declare that they have no competing interests.

## Authors’ contributions

BKH analyzed and interpreted the patient data regarding the hematological disease and was a major contributor in writing the manuscript. AM analyzed and interpreted the patient data regarding the neurological disease. LMK performed the microscopic examination of bone marrow. WJ, AA, EAD and AB analyzed and interpreted the patient data regarding the hematological disease. IR performed the microscopic examination of bone marrow. All authors read and approved the final manuscript.
